# Dietary Polyphenols as Modulators of *Bifidobacterium* in the Human Gut Microbiota

**DOI:** 10.3390/nu18050782

**Published:** 2026-02-27

**Authors:** Corina Dalia Toderescu, Mohamudha Parveen, Svetlana Trifunschi, Alexandru Oancea, Gentiana Camelia Ciubuc (Jurj), Iulia Gabriela Cresneac, Melania Florina Munteanu, Ioana Ciopanoiu, Casiana Boru, Elena Narcisa Pogurschi, Catalin Ionite, Alina Stefanache, Ionut Iulian Lungu

**Affiliations:** 1 Faculty of Pharmacy, Western University “Vasile Goldis” Arad, 310048 Arad, Romania; toderescu.corina@uvvg.ro (C.D.T.); cresneac.iulia@uvvg.ro (I.G.C.); munteanu.melania@uvvg.ro (M.F.M.); ciopanoiu.ioana@uvvg.ro (I.C.); 2 School of Life and Health Science, University of Roehampton, London SW154JD, UK; mohamudha.parveen@roehampton.ac.uk; 3 Faculty of F Medicines, Western University “Vasile Goldis” Arad, 310048 Arad, Romania; boru.casiana@uvvg.ro; 4 Faculty of Animal Productions Engineering and Management, University of Agronomic Sciences and Veterinary Medicine of Bucharest, 59 Marasti Blvd, District 1, 011464 Bucharest, Romania; elena.pogurschi@usamv.ro; 5 Grigore T. Popa University of Medicine and Pharmacy Iasi, 700115 Iasi, Romaniaalina.stefanache@umfiasi.ro (A.S.); ionut-iulian.lungu@umfiasi.ro (I.I.L.)

**Keywords:** dietary polyphenols, gut microbiota, *Bifidobacterium*, metabolic health

## Abstract

Background: Polyphenols—bioactive compounds abundant in plant-based foods—are increasingly recognised for their capacity to modulate the gut microbiota. As the gut microbiome plays a central role in metabolic regulation, immune function, and disease prevention, understanding how specific polyphenol subclasses influence microbial diversity and functionality remains essential. Despite growing evidence of their benefits, the precise effects of flavonoids, phenolic acids, and anthocyanins on gut microbial composition are not yet fully clarified. Objective: This study aimed to evaluate the impact of dietary polyphenols on gut microbiota composition and function, with a particular focus on the abundance of *Bifidobacterium*, a key beneficial genus associated with metabolic and immune health. It was hypothesised that polyphenol-rich interventions were associated with increases in *Bifidobacterium* abundance and enhance overall microbial diversity. Design: A systematic review and meta-analysis were conducted following PRISMA guidelines. Human intervention studies published between January 2015 and February 2025 were retrieved from PubMed, Scopus, and Web of Science. A predefined PICO framework guided study selection. Twenty-two studies were synthesised using thematic analysis, and four of these were eligible for quantitative meta-analysis. The meta-analysis was performed in R (version 4.4.1) using the metafor and meta packages, calculating standardised mean differences (SMD) under a random-effects model to account for heterogeneity. Extracted data included study design, population characteristics, polyphenol subclass, intervention type, microbiome assessment method, and key outcomes. Results: Across the 22 reviewed studies, polyphenols—particularly flavonoids and phenolic acids from foods such as berries, grape pomace, and green tea—consistently increased beneficial microbial taxa including *Bifidobacterium*, *Faecalibacterium*, and *Lactobacillus*. These microbial shifts were associated with improved metabolic markers, reduced inflammation, and enhancements in gut barrier integrity. Polyphenol-rich dietary patterns also showed benefits in conditions such as NAFLD, prediabetes, and depression. However, findings were influenced by interindividual variability, short intervention durations, and inconsistent methodologies. The meta-analysis revealed a significant positive effect of polyphenol intake on *Bifidobacterium* abundance (SMD = 0.81; 95% CI: 0.18–1.44; *p* = 0.0114), corresponding to a moderate-to-large effect size. Substantial heterogeneity (*I*^2^ = 77.4%) suggested considerable variation in intervention types, dosage, study design, and microbiome analysis methods. Conclusions: Polyphenol-rich diets were associated with increased *Bifidobacterium* abundance and favourable modulation of gut microbiota composition, supporting their potential as a nutritional strategy to enhance gut and metabolic health. However, interstudy variability highlights the need for more standardised, long-term, and mechanistically focused human trials. Future research should incorporate multi-omics approaches, personalised nutrition frameworks, and consistent microbiome analysis methods to better understand the pathways linking polyphenol intake and host health outcomes.

## 1. Introduction

In recent years, the intricate relationship between diet and gut microbiota has emerged as a central focus in nutritional and biomedical research. The gut microbiota—a highly diverse and metabolically active ecosystem of trillions of microorganisms residing in the human gastrointestinal tract—plays a pivotal role in regulating numerous physiological processes including nutrient absorption, immune system modulation, maintenance of intestinal barrier integrity, vitamin synthesis, and defense against pathogens [1]. Dysbiosis, an imbalance or alteration in this microbial community, has been increasingly associated with a range of disorders such as inflammatory bowel disease (IBD), obesity, type 2 diabetes, cardiovascular disease, colorectal cancer, and even neurological and mood disorders through the gut–brain axis. Consequently, understanding how specific dietary components influence the composition and activity of gut microbiota has become a scientific and clinical priority.

Among the many dietary factors, polyphenols—naturally occurring secondary metabolites widely present in plant-based foods such as fruits, vegetables, tea, coffee, red wine, whole grains, chocolate, and nuts—have received growing attention for their potential to modulate gut microbial balance. These compounds are some of the most abundant antioxidants in the human diet and are structurally diverse, affecting their biological activity and bioavailability. Broadly, polyphenols are classified into two main groups: flavonoids (including quercetin, catechins, and anthocyanins) and non-flavonoids (such as phenolic acids, stilbenes like resveratrol, and lignans) [2]. Their known antioxidant and anti-inflammatory properties contribute to the prevention of chronic diseases including cardiovascular disorders, diabetes, metabolic syndrome, and certain cancers.

Protective effects against chronic diseases. Emerging evidence indicates that diets rich in polyphenols are associated with improvements in cardiometabolic health through reductions in blood pressure, cholesterol levels, and endothelial dysfunction—effects partly mediated by gut microbial metabolites Furthermore, the modulation of gut microbiota by polyphenols has been linked to neuroprotective benefits, including reduced neuroinflammation and improved cognitive resilience via the gut–brain axis. Thus, dietary polyphenols not only act as antioxidants but also as prebiotic-like agents that contribute to microbial homeostasis and overall well-being.

Despite substantial progress, significant research gaps remain. Much of the existing evidence on polyphenol–microbiota interactions derives from in vitro and animal studies, while well-controlled human clinical trials are still limited [3]. Moreover, interindividual variability in microbiota composition leads to differential metabolic responses to polyphenol intake, complicating the generalization of results and the establishment of precise dietary recommendations. The growing burden of non-communicable, diet-related diseases worldwide underscores the need for translational human research exploring how polyphenols modulate microbial diversity and A unique aspect of polyphenol biology is their limited bioavailability in the upper gastrointestinal tract. Due to their complex chemical structures and poor absorption in the small intestine, a significant proportion of ingested polyphenols reaches the colon intact, where they come into direct contact with the gut microbiota [4]. Within this environment, gut microorganisms extensively metabolize polyphenols into smaller, bioactive compounds such as urolithins and phenylacetic acids, which often display enhanced physiological activity. This metabolic interplay is bidirectional: while the gut microbiota transforms and determines the bioavailability and activity of polyphenols, these compounds in turn selectively influence the composition and function of the microbial ecosystem.

Polyphenols act as modulators of the gut microbiota by promoting the growth of beneficial bacterial taxa such as *Lactobacillus*, *Bifidobacterium*, *Faecalibacterium*, and *Akkermansia*, while inhibiting potentially pathogenic species including *Clostridium difficile* and *Escherichia coli* [5]. Moreover, polyphenols can enhance the production of short-chain fatty acids (SCFAs)—including butyrate, propionate, and acetate—through microbial fermentation. SCFAs play essential signaling roles in maintaining intestinal barrier function, regulating inflammation, appetite, and glucose homeostasis [6]. These beneficial microbial and metabolic shifts contribute to improved systemic outcomes such as reduced inflammation, enhanced insulin sensitivity, and better lipid metabolism.

The polyphenol–microbiota axis represents a critical mechanism by which diet exerts metabolic function [7].

The aim of this study is therefore to investigate how dietary polyphenols influence the composition and functional dynamics of the human gut microbiota, and to elucidate the mechanisms linking these interactions to metabolic and inflammatory pathways. Specifically, the study seeks to:

1. Examine how different classes of polyphenols modulate gut microbial communities;
2. Elucidate the mechanisms through which these interactions affect metabolic pathways and microbial diversity;
3. Assess the effects of polyphenol intake on gut dysbiosis-related disorders; and
4. Identify key limitations and future directions in current polyphenol–microbiome research

## 2. Materials and Methods

### 2.1. Study Design

This study employed a systematic review approach, incorporating both qualitative synthesis and, where appropriate, meta-analytical elements, to comprehensively evaluate the influence of dietary polyphenols on gut microbiota composition and function. The design was structured to ensure methodological rigor, transparency, and reproducibility, in alignment with the Preferred Reporting Items for Systematic Reviews and Meta-Analyses [8] (PRISMA 2020) guidelines [8] (Page et al., 2021). The systematic review framework enables the rigorous identification, evaluation, and synthesis of existing evidence, providing a high level of reliability for summarizing the current state of research. Where applicable, a meta-analytic approach was considered to quantify pooled effect sizes, recognizing that statistical integration enhances the interpretability of heterogeneous datasets [9].

Given the diversity in polyphenol structures, dietary sources, intervention protocols, and microbiota assessment methodologies, a qualitative evidence synthesis was prioritized to capture mechanistic and compositional insights that may not lend themselves to strict statistical aggregation [10]. This thematic synthesis approach allows for the inclusion of studies employing various analytical platforms such as 16S rRNA sequencing, metagenomics, and metabolomics, facilitating a more comprehensive understanding of how polyphenols modulate microbial diversity, abundance, and metabolite production—particularly short-chain fatty acids (SCFAs), which are key indicators of gut microbial functionality.

The study protocol and objectives were pre-defined to minimize bias and enhance reproducibility. Searches were limited to human intervention studies published January 2015 and February 2025, as these provide the most direct evidence for translational relevance. Both healthy individuals and subjects with metabolic or gut-related disorders (such as obesity or non-alcoholic fatty liver disease) were included to capture variability in microbial responses to polyphenol intake. Studies involving animal or in vitro models were excluded unless they provided critical mechanistic insights that supported human data.

The review design was further guided by PICO (Population, Intervention, Comparison, Outcome) framework, which is underlined in Table 1, to ensure clarity and consistency in study selection, screening, and data extraction. This structured approach facilitated systematic inclusion of relevant studies and comparability across diverse research contexts.

Results derived from DGGE and targeted qPCR were interpreted with caution and treated as complementary methodological outputs, rather than directly comparable to community-wide diversity estimates obtained through 16S rRNA amplicon sequencing or metagenomic approaches.

By combining both qualitative thematic analysis and quantitative synthesis, this design ensures a holistic understanding of how polyphenols interact with the gut microbial ecosystem. It also addresses inconsistencies across individual studies, which often arise due to differences in study populations, polyphenol sources, dosages, and analytical methodologies. Overall, the systematic and integrative nature of this design provides a robust foundation for identifying consistent microbial patterns attributable to polyphenol consumption and for elucidating the mechanisms underpinning these effects.

### 2.2. Keyword Search Strategy

A comprehensive and systematic literature search was conducted across three major electronic databases: PubMed, Scopus, and Web of Science, chosen for their extensive coverage of biomedical, nutritional, and microbiome-related research. This multi-database approach ensured broad inclusion of studies from diverse disciplines, including clinical nutrition, molecular biology, and human health interventions. The search was designed to identify relevant studies published between January 2015 and February 2025, reflecting the most current evidence in this rapidly evolving field.

To capture a wide and inclusive range of literature, the search strategy employed a combination of Medical Subject Headings (MeSH) and free-text terms, linked using Boolean operators (“AND,” “OR”) to refine the query structure. Table 2 highlights primary search terms included:

“polyphenols” OR “dietary polyphenols” OR “phenolic compounds” OR “flavonoids” OR “phenolic acids”

AND

“gut microbiome” OR “gut microbiota” OR “intestinal flora” OR “microbial composition” OR “microbiota diversity”

AND

“human”.

These terms were selected to encompass the major classes of polyphenols (such as flavonoids, stilbenes, lignans, and phenolic acids) and the key descriptors of gut microbial ecology. Where applicable, database-specific MeSH and keyword mapping were used to maximize retrieval sensitivity and specificity.

To ensure the inclusion of original research articles with direct empirical data, several filters were applied. The search was restricted to studies that met the following criteria:

- Full-text availability in English language;
- Human studies only;
- Original research articles reporting quantitative or qualitative outcomes on gut microbiota composition, microbial diversity, or related metabolic biomarkers (e.g., short-chain fatty acids);
- Exclusion of review articles, systematic reviews, meta-analyses, editorials, and commentaries.

Additionally, a manual screening of the reference lists of all included studies and relevant reviews was performed to identify any additional articles that may have been missed through database searching. This snowballing technique enhanced the comprehensiveness of the search and minimized publication bias by incorporating potentially overlooked but relevant literature.

The search strategy and inclusion criteria were developed and reviewed in accordance with PRISMA 2020 guidelines [8] to maintain transparency, reproducibility, and methodological rigor. All retrieved records were imported into a citation management software for systematic screening, duplicate removal, and further eligibility assessment.

### 2.3. Eligibility Criteria (PICO Framework and Study Selection)

Establishing clear eligibility criteria was essential to ensure the relevance, methodological rigor, and comparability of the studies included in this systematic review. The criteria were defined using the PICO (Population, Intervention, Comparator, Outcome) framework and complemented by specific inclusion and exclusion parameters to guide the selection process appear in Table 3. This dual framework allowed for a transparent, structured, and reproducible approach to identifying studies relevant to the effects of dietary polyphenols on gut microbiota composition.

The PICO structure ensured that only studies directly examining the relationship between polyphenol intake and gut microbiota composition in human populations were considered for inclusion.

#### Inclusion and Exclusion Criteria

In addition to the PICO framework, Table 4 show explicit inclusion and exclusion criteria were established to maintain consistency, quality, and focus across the selected studies. These parameters were aligned with PRISMA 2020 recommendations for systematic reviews [8].

This structured eligibility framework ensured that all included studies provided robust, human-based evidence on the modulatory effects of dietary polyphenols on gut microbial ecology. The combination of PICO-based conceptual clarity and strict inclusion/exclusion criteria strengthened the validity of the synthesis by filtering out studies with methodological limitations, incomplete data, or irrelevant outcomes.

In the context of this review, polyphenol intervention was considered to be one of the dietary exposures whereby the major experimental difference could be ascribed to polyphenols, either as (i) polyphenol-rich whole foods (e.g., berries, cocoa, tea, grapes) with quantifiable or well-defined polyphenol profiles, or (ii) supplements/extracts of specific polyphenolic compounds or total confirmed polyphenols. Mixed diets (e.g., Mediterranean-style diets) were also eligible provided it was clear that the intervention specifically focused on the presence of polyphenol-rich foods and an exposure marker (e.g., quantified polyphenol intake, total polyphenols or characterised polyphenol sources) was reported on the basis of which the effects of gut-microbiota could be attributed to the change in dietary polyphenols. Interventions mainly mediated by non-polyphenol bioactives, isolated fibre/prebiotic interventions, probiotics, omega-3 fatty acids and sulforaphane interventions were not counted as polyphenol interventions unless polyphenols was the active ingredient and reported separately.

*Bifidobacterium* is an interesting taxon in terms of its beneficial effects on dietary factors as it is often linked to the protective effect of the gut barrier, immunomodulatory activity, and cross-feeding that facilitates the production of short-chain fatty acids. In turn, explaining the consistent increase in *Bifidobacterium* by polyphenol-rich interventions, and in what circumstances, is the key to applying the results of polyphenol and microbiota interactions to the dietary recommendations.

### 2.4. Study Selection and Implementation of Criteria

The study selection and screening process adhered to a structured, transparent, and unbiased protocol in line with the Preferred Reporting Items for Systematic Reviews and Meta-Analyses [8] (PRISMA 2020) guidelines [8]. A two-step manual screening approach was implemented to ensure methodological rigor and consistency throughout the selection process [11]. Automated eligibility screenings were used only to remove obviously irrelevant records (e.g., non-human studies, non-intervention format, or non-research item) and all full-text eligibility decisions were then checked manually against the used PICO criteria.

Step 1: Title and Abstract Screening

All records retrieved from the database searches (PubMed, Scopus, and Web of Science) were initially imported into a reference management software for deduplication. Out of 261 records initially identified, 200 articles remained after removal of duplicates and were screened based on titles and abstracts. Each record was independently assessed by reviewers to determine its relevance against the predefined inclusion and exclusion criteria (outlined in Section 2.3). Studies that appeared relevant or lacked sufficient detail in their abstracts were advanced to the next stage for full-text evaluation.

Step 2: Full-Text Review and Eligibility Assessment

During full-text screening, each study was critically evaluated for compliance with the eligibility standards. This step involved independent assessments by reviewers to reduce selection bias and ensure objectivity. A smaller group of full-text articles met the eligibility requirements subsequent to this step. After full-text validation and risk-of-bias assessment (Section 2.6), 10 studies passed the inclusion criteria of the final synthesis, and six of them became a narrative synthesis and four of them gave data suitable to be included in a meta-analysis. The screening procedure was done twice at the title/abstract and full-text phases. Systematic exclusion reasons upon reaching the full-text review were documented (e.g., inappropriate population, non-interventional study design, inadequate reporting of the microbiome outcomes or inadequate extractable data to synthesize). This will eliminate the need to guess the basis of the eligibility criteria and the resulting list of the included studies.

Step 3: Inclusion Parameters for Final Selection

Studies were only included in the final synthesis if they clearly specified the following methodological components:

- Defined polyphenol dose and source (e.g., food-based or supplemental origin)
- Duration of the dietary intervention
- Microbiota analysis method employed (e.g., 16S rRNA sequencing, metagenomics)
- Quantifiable outcomes directly related to microbial diversity or compositional shifts

These parameters ensured methodological comparability across studies and enhanced the reliability of the synthesized findings.

Step 4: Documentation and Transparency

The entire selection process was documented and visually summarized using a PRISMA flow diagram, detailing the number of studies identified, screened, excluded (with specific reasons), and ultimately included in the final review. This transparent reporting structure reinforces the reproducibility and credibility of the review methodology.

By systematically implementing these steps, the study ensured that only the most relevant, high-quality evidence was synthesized, minimizing the risk of bias and maximizing the robustness of conclusions regarding the influence of dietary polyphenols on gut microbiota composition.

### 2.5. Ethical Considerations

As this study is based on a systematic review and meta-analysis of previously published research, there was no direct involvement of human or animal participants; therefore, formal ethical approval was not required. Nonetheless, the selection of studies adhered to ethical standards by including only research that had obtained appropriate ethical approval from the respective institutions [12]. All data utilized were sourced from publicly available, peer-reviewed articles and were presented accurately, without fabrication or misrepresentation. Throughout the research process, proper citation practices and adherence to academic integrity were maintained to ensure transparency, accountability, and respect for the original authors’ contributions.

### 2.6. Presentation of Risk of Bias and Study Quality

The individual-study level was used to measure risk of bias to be able to interpret both the systematic synthesis and the subsequent meta-analysis. Randomised controlled trials were assessed based on the Cochrane Risk of Bias tool (RoB2), which reviews bias due to the approach of randomisation and departure of the planned interventions, missing outcome data, evaluation of outcome and reporting of the outcome of the study. In the case of non-randomised intervention studies, a more suitable structured appraisal framework was used, which focused on the issue of confounding, selection bias, intervention classification, missing data, outcome measures, and selective reporting. Quality appraisals were done by two reviewers and whenever there was a disagreement, it was discussed. The soundness of the conclusions was then put into perspective by risk-of-bias judgement to be used in sensitivity analysis in the quantitative synthesis as needed.

## 3. Results

### 3.1. Study Selection

**Figure 1:**
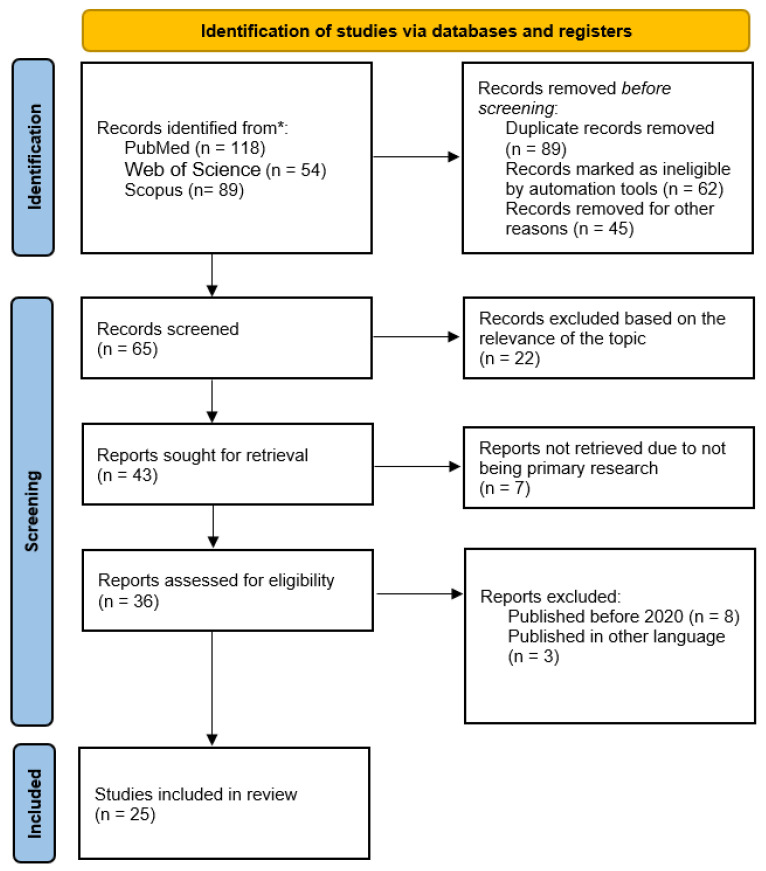
PRISMA flowchart of studies selection. (Source: Self-made). * = specify all information sources.

PRISMA flow diagram (Figure 1) is a summary of the study identification and selection process. PubMed (n = 118), Scopus (n = 89), and Web of Science (n = 54) were searched to retrieve a total of 261 records. Before the screening process, 89 duplicates were accepted, 62 records were not accepted in the case of automated eligibility screening, and 45 records were not accepted due to other reasons, including 65 records that were eligible to undergo title/abstract screening. Among them 22 records were excluded because of their irrelevance, and 43 reports were identified to be retrieved in their entirety. The number of reports that could not be accessed due to not being primary research was seven, which resulted in the remaining number of 36 full-text reports that could be evaluated as eligible. Eleventh full-text reports were eliminated (published before 2020, n = 8; published in another language, n = 3). Finally, 22 articles were eligible and included in the systematic review. In the case of a meta-analysis, the subset of the studies that had sufficiently comparable and extractable *Bifidobacterium* outcome data were pooled, and the rest of them entered the qualitative synthesis.

### 3.2. Characteristics of Included Studies

To meet the eligibility criteria in Section 2.3, the following table (Table 5), labeled Characteristics of Included Studies, has been made to distinguish (a) the case of human dietary intervention studies that were included in the systematic synthesis, and (b) the contextual evidence (i.e., narrative or review articles) used to make mechanistic interpretations but not constituted included intervention evidence. Only the human intervention trials that met the criteria of the PICO framework were included in the PRISMA counts and the ultimate synthesis.

### 3.3. Data Analysis

#### 3.3.1. Findings of the Systematic Review

- Modulation of Gut Microbiome by Polyphenol Classes

In the reviewed literature, various classes of polyphenols—including flavonoids, phenolic acids, stilbenes, proanthocyanidins, and anthocyanins—frequently reported shown to influence gut microbiota composition by promoting the growth of beneficial microbes and inhibiting potentially harmful ones. Evidence from intervention trials [5,13,14,15,20] demonstrated that consumption of polyphenol-rich foods such as red raspberries, blueberries, aronia berries, and grape extracts led to notable increases in beneficial bacterial populations, including *Bifidobacterium*, *Faecalibacterium*, *Anaerostipes*, and *Butyricimonas faecihominis*. Similarly, flavonoid-rich orange juice [31] and wild blueberry powder [20] were associated with greater abundance of *Lachnospiraceae* and *Roseburia*, taxa involved in short-chain fatty acid (SCFA) production. Anthocyanin-rich apples [16] increased *Lactobacillus* while reducing *Ruminococcus*, suggesting a rebalancing of the microbial community toward a more health-promoting profile. Additionally, polyphenols from olive oil, green tea, and dark chocolate enhanced *Clostridium leptum* and overall microbial diversity, while decreasing *Eubacterium rectale–Blautia coccoides* in individuals with cardiometabolic risk [21]. Collectively, these findings suggest that distinct polyphenol types modulate gut microbial communities in specific ways, supporting the concept that dietary polyphenols are key regulators of microbiota composition and metabolic health [25,26].

b. Influence on Metabolic Pathways and Microbial Diversity

Microbial fermentation and the resulting production of bioactive metabolites underpin the metabolic influence of polyphenols on the host. Supplementation with polyphenol-rich foods and extracts has been shown to enhance microbial gene richness and modulate functional pathways linked to host metabolism. For example, Aronia extract increased microbial diversity and upregulated pathways related to propionate production and GABA biosynthesis [15] while blueberry polyphenols promoted short-chain fatty acid (SCFA) formation and antioxidant capacity, particularly among older adults [13,20] observed metabolomic alterations—such as changes in serum amino acids and bile acids—without major shifts in microbial composition, suggesting that polyphenols may exert functional rather than structural modulation of the gut microbiota.

Consistently, polyphenol-rich diets have been positively associated with improved cardiometabolic biomarkers and enhanced microbial diversity. Diets enriched with walnuts, green tea, and Mankai within a Green-Mediterranean (Green-MED) framework increased *Prevotella* abundance [17] and branched-chain amino acid degradation [24,27]. Similarly, individuals with obesity or metabolic syndrome adhering to Mediterranean-style diets demonstrated increased gene richness and elevated levels of *Faecalibacterium prausnitzii* [27]. Diets rich in polyphenols and long-chain omega-3 fatty acids further enhanced microbial diversity—particularly in the *Clostridium leptum* group—and improved insulin secretion [21]. Complementing these findings, the MaPLE trial [29] revealed that cocoa and green tea polyphenols not only altered the serum metabolome but also promoted SCFA-producing bacteria such as *Butyricicoccus* and *Faecalibacterium*, shifting the microbial ecosystem toward a higher SCFA-producing profile while reducing SCFA-degrading taxa. Together, these results highlight the capacity of dietary polyphenols to shape gut microbial functionality and metabolic health through complex, multi-level interactions between microbial metabolism and host physiology. Both within-sample (alpha) and between-sample (beta) diversity were measured using diversity metrics based on high-throughput sequencing (e.g., Shannon index, observed amplicon sequence variants [ASVs], operational taxonomic units [OTUs], gene richness]) and dissimilarity measures (e.g., weighted and unweighted UniFrac, Bray–Curtis). The changes in microbial richness and community structure during the intervention of polyphenol were assessed using these indices.

c. Impact on Gut Dysbiosis and Related Disorders

Polyphenols have demonstrated considerable therapeutic potential in correcting gut dysbiosis associated with various non-communicable and metabolic diseases. Evidence suggests that their beneficial effects are mediated through modulation of the gut microbiota, leading to systemic health improvements. For instance, litchi-derived oligonol was shown to suppress hepatic steatosis while promoting beneficial microbes such as *Akkermansia* and *Dialister* in patients with non-alcoholic fatty liver disease [30]. Similarly, dietary interventions using broccoli sprout extract [32], postbiotics [33] and red raspberries [6] in prediabetic adults significantly reduced fasting glucose, insulin resistance, and HbA1c levels by modulating gut microbial composition and activity.

In the cardiometabolic domain, *Clostridium leptum* (CLEPT)-associated microbial shifts induced by polyphenol intake were linked to improved early insulin secretion [21]. Moreover, *Aronia* polyphenols improved vascular function through enhanced flow-mediated dilation, with microbial changes correlating to favorable cardiovascular biomarkers [34]. These findings reinforce the role of polyphenol-induced microbial modulation in promoting cardiometabolic health.

Beyond metabolic outcomes, polyphenols have also exhibited effects on neurological and inflammatory pathways. [31] reported that supplementation with fermentable flavonoids reduced depressive symptoms by increasing *Lachnospiraceae* abundance, supporting the gut–brain axis hypothesis. Onali et al. (2025) [18] demonstrated that berry supplementation protected against meat-induced dysbiosis and colon carcinogenesis, while [28] highlighted the neuroprotective potential of polyphenols in multiple sclerosis through anti-inflammatory and microbiota-regulating actions. Collectively, these studies emphasize that dietary polyphenols, via their capacity to restore microbial balance, may serve as promising adjunctive agents in the prevention and management of metabolic, cardiovascular, and neuroinflammatory disorders.

d. Research Gaps and Future Advancements

While current evidence highlights the promising potential of dietary polyphenols in modulating the gut microbiota, several limitations and knowledge gaps remain. Many studies, including those by [20,22], have reported negligible or non-significant microbiota changes—likely due to short intervention durations, small sample sizes, or the limited sensitivity of sequencing and analytical techniques. Furthermore, as [10] emphasized, individual metabotypes—distinct microbial and metabolic profiles influencing polyphenol metabolism and efficacy—play a crucial role, underscoring the need for more personalized nutrition approaches.

Another major gap lies in understanding species-level microbial interactions and the long-term persistence of microbiome shifts following sustained polyphenol intake. Additionally, much of the existing research has focused on taxonomic rather than functional microbial changes, restricting insights into mechanistic pathways. Future investigations should therefore prioritize multi-omics approaches—integrating metagenomics, metabolomics, and transcriptomics—to unravel the complex host–microbe–nutrient interactions, as also suggested by Peron et al. [29].

To enhance comparability and generalizability, standardized polyphenol dosages and methodologies must be adopted in well-controlled, long-term trials involving diverse populations across age, sex, and ethnicity. Studies such as Dueñas et al. (2015) [35] and Meslier et al. (2020) [27]. already offer valuable proof of concept, demonstrating that the identification of gut microbial predictors of dietary response can effectively guide targeted, polyphenol-based nutritional interventions.

#### 3.3.2. Microbiome and Functional Readouts That Are Common to Inclusion Studies

The microbiota outcome measures used in the intervention trials included were in varied methodological breadth and analytic depth. The degree of community composition and diversity was measured through 16S rRNA gene amplicon sequencing, which allows evaluation of alpha- and beta-diversity indices, including Shannon, Simpson and Bray–Curtis dissimilarity. Shotgun metagenomic sequencing provided species-level resolution and functional potential enabling the taxonomic characterization to the species level and gene family annotation of metabolic pathways. Quantitative PCR of conserved gene markers gave absolute/relative abundance of chosen taxa (e.g., *Bifidobacterium* spp.), targeted and untargeted metabolomics platforms gave a functional biomarker (e.g., short-chain fatty acids (SCFA), bile acids and polyphenol-derived metabolites). The variation in the methodological choice was treated as a major contributor of variability in the synthesis of the results across studies.

#### 3.3.3. Findings for Meta-Analysis

**Figure 2:**
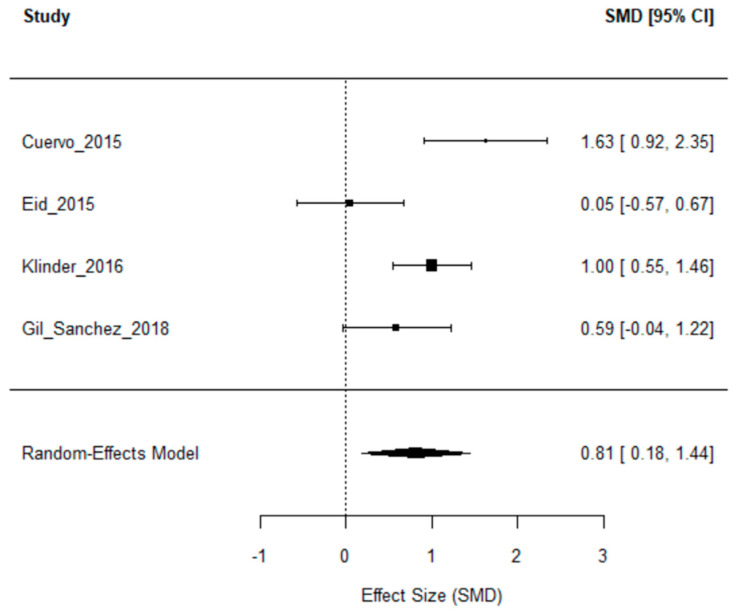
Meta-analysis—forest plot. (Source: Self-created.). Square—individual study effect sizez with size proportional to study weight; Horizontal lines—95% confidence intervals; Vertical dashed line—no effect (SMD = 0); Diamond—pooled effect estimate from the rendom-effects model.

To evaluate the impact of dietary interventions on *Bifidobacterium* levels in humans, this meta-analysis synthesized findings from four studies [36,37,38,39]. The meta-analysis, Figure 2., included only human intervention studies in which the primary exposure was polyphenol-rich foods or defined polyphenol supplements and where extractable *Bifidobacterium* data were available. Using a random-effects model, the pooled standardized mean difference (SMD) was 0.81 (95% CI: 0.18–1.44, *p* = 0.0114), indicating a moderate to large overall effect of dietary interventions on *Bifidobacterium* abundance.

**Figure 3:**
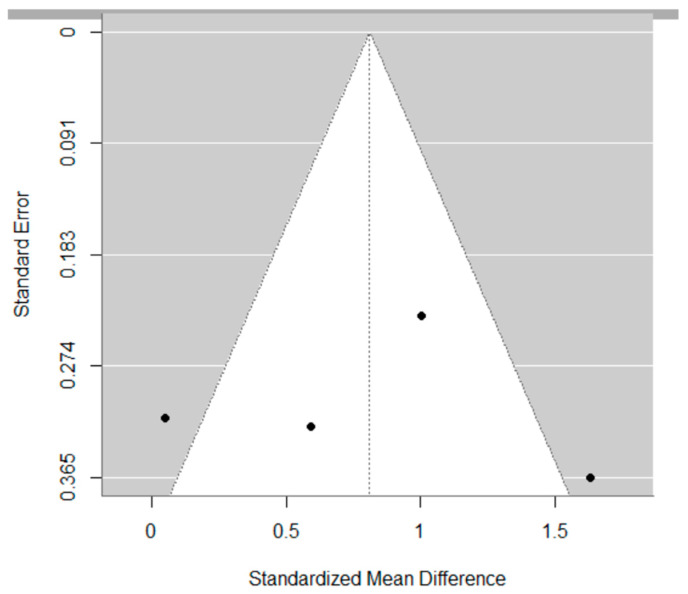
Meta Analysis—Funnel Plot. (Source: Self-created.). Each dot represent an individual study plotted by standardized mean difference and standard error; Grey represent 95% confidence limit around the pooled estimate.

However, substantial heterogeneity was detected (*I*^2^ = 77.4%; *Q*(3) = 12.03, *p* = 0.0073), likely reflecting variability in study designs, intervention types, durations, and microbiota assessment methods as the Figure 3. shows. For instance, both [37,38] reported significant increases in *Bifidobacterium* following grape polyphenol and fibre supplementation, respectively, whereas [37] observed only minor changes, and [37] provided limited statistical detail, which may have affected the precision of the pooled estimate.

Where necessary, missing standard deviations were estimated using literature averages, a step that may have introduced some bias. Funnel plot inspection was performed; however, given the small number of included studies (n = 4), publication bias could not be reliably assessed. Overall, these results suggest that polyphenol-rich dietary interventions may enhance *Bifidobacterium* abundance, although interpretation should be cautious due to heterogeneity and limited sample size.

## 4. Discussion

### 4.1. Summary of Key Findings

Results from this study generally indicate that dietary polyphenols—particularly flavonoids, phenolic acids, and anthocyanins—exert beneficial regulatory effects on the gut microbiome. Across the reviewed evidence, polyphenol-rich foods such as red raspberries, blueberries, apples, and green tea were associated with increased abundance of key beneficial bacterial genera, including *Bifidobacterium*, *Anaerostipes*, *Faecalibacterium prausnitzii*, *Lactobacillus*, and *Akkermansia* [5,13,16,17,21,34]. Beyond structural changes in microbial populations, polyphenol intake was positively correlated with greater microbial diversity, enrichment of metabolic pathways such as GABA biosynthesis, and improvements in cardiometabolic health markers, including enhanced SCFA production and increased microbial gene richness [14,15,24,27]. The meta-analysis examining *Bifidobacterium* abundance (n = 4) demonstrated a significant pooled standardised mean difference of 0.81 (95% CI: 0.18–1.44, *p* = 0.0114), indicating a moderate-to-large effect of dietary interventions on this beneficial genus. However, considerable heterogeneity (*I*^2^ = 77.4%; *Q*(3) = 12.03, *p* = 0.0073) suggests that differences in study design, intervention types, durations, and microbiota assessment methods contributed to variability in outcomes. Overall, the evidence supports the potential of polyphenol-rich diets not only to modulate the gut microbiota but also to contribute to improved outcomes across several conditions, including NAFLD, prediabetes, depression, and multiple sclerosis [28,30,31]. Nevertheless, key research gaps remain, including interindividual variability in responses, limited study durations, and insufficient species-level resolution. Future work should incorporate multi-omics approaches—such as metagenomics, metabolomics, and transcriptomics—and adopt standardized protocols to advance personalized and clinically applicable polyphenol-based interventions [10,29].

The strength of evidence in respect of *Bifidobacterium* depends on the interventional comparability and the fineness of measuring instruments. Some of these studies used specific assays (including quantitative polymerase chain reaction) but others used sequencing-derived relative abundance metrics, which do not in any way form the basis of each other; therefore, the derived conclusions were deliberately conservative and framed with reference to the limitations of the platform and inherent variability in the baseline microbiota.

Polyphenols can be utilised selectively by bacteria that can metabolise polyphenol structures, leading to the production of smaller phenolic metabolites that, in turn, control the ecology of the community by substrate competition, cross-feeding and environmental modification (such as redox effects). Practically, this would benefit taxa associated with saccharolytic fermentation and SCFA production and at the same time inhibit the growth of pathobionts in dysbiosis-prone environments. Notably, there are class and baseline microbiota-specific responses to polyphenols (so-called metabotype), and this observation explains why some trials see stronger *Bifidobacterium* increases than others.

### 4.2. Comparison with Previous Research

These findings align closely with a growing body of evidence demonstrating that dietary polyphenols exert beneficial effects on gut microbiota composition. For example, Kalt et al. (2019) [40] reported that blueberry supplementation increased the abundance of beneficial gut microorganisms and improved cardiovascular markers in older adults. Similar outcomes have been observed in interventions using other polyphenol-rich foods, including cocoa, green tea, berries, and red wine, which consistently promote the growth of beneficial bacteria such as *Bifidobacterium* and *Lactobacillus* while suppressing potentially pathogenic taxa [40,41,42].

More recent studies further reinforce these findings. For instance, Anhê et al. (2015) [43] and Roopchand et al. (2015) [44] demonstrated that polyphenol-rich extracts modulated gut microbial composition and improved metabolic outcomes, partly through enrichment of *Bifidobacterium* spp. Likewise, Ma and Chen (2020) [45] reported significant increases in *Lactobacillus* and *Bifidobacterium* alongside reductions in *Clostridium* species following polyphenol supplementation. Comparable microbial shifts have also been reported in human intervention studies published over the last decade, including those examining berry-derived anthocyanins, tea catechins, and mixed polyphenol formulations [35,46,47].

These observations are consistent with the findings of the current meta-analysis, which identified a significant pooled standardised mean difference (SMD) of 0.81 (95% CI: 0.18–1.44, *p* = 0.0114) in favour of polyphenol interventions, indicating a moderate-to-large positive effect on *Bifidobacterium* abundance. Importantly, despite heterogeneity in polyphenol source, dosage, and intervention duration across studies, the directionality of microbial responses—particularly *Bifidobacterium* enrichment—remains remarkably consistent.

Collectively, evidence from controlled dietary interventions, mechanistic studies, and the aggregated findings of this meta-analysis supports the hypothesis that dietary polyphenols may play a meaningful role in modulating gut microbiota composition, thereby supporting the outcomes presented in this review.

### 4.3. Strengths

This systematic review and meta-analysis provides a robust and methodologically rigorous evaluation of how different polyphenol subclasses influence the gut microbiota [9]. A major strength is the clear, comprehensive, and transparent methodology, supported by strict adherence to PRISMA guidelines, which enhances reproducibility and reliability [8]. The use of a well-defined PICO framework ensured a focused selection of human studies published between January 2025 and February 2025, strengthening the clinical relevance of the findings. Statistically, the application of the standardised mean difference (SMD) as the effect size metric and the implementation of a random-effects model with Restricted Maximum Likelihood (REML) estimation improved the robustness of quantitative synthesis. In addition, the thematic analysis provided structured interpretation of diverse study outputs, facilitating clearer insights into microbiome modulation, metabolic pathways, and disease associations, compared with unstructured reporting across studies.

### 4.4. Limitations

Despite its strengths, this review has several limitations. Only four studies were eligible for inclusion in the meta-analysis, leading to high heterogeneity (*I*^2^ = 77.4%), likely arising from differences in study designs, intervention types, durations, and microbiota assessment methods [46,48]. The small number of available randomized controlled trials and the reliance on published English-language data introduce risks of both language and publication bias, reducing the generalizability of the findings [48,49,50,51]. Methodological inconsistencies—such as variation in polyphenol dosages, intervention lengths, and sequencing techniques—further complicate cross-study comparisons [52,53]. In several cases, missing standard deviations were estimated using literature averages, which may introduce bias into the pooled effect sizes [51]. More broadly, the lack of long-term population-level studies and insufficient individual-level microbiome data hinder conclusions about sustained microbiome shifts and personalized responses to polyphenol intake [54,55]. Additionally, distinctions between the effects of specific polyphenol subclasses (e.g., anthocyanins vs. other types) are still insufficiently catalogued, underscoring the need for future studies employing standardized, multi-omics approaches to strengthen mechanistic and clinical insights [35,47,53].

### 4.5. Implications for Practice and Future Research

The findings of this review suggest that dietary polyphenols—particularly those rich in flavonoids and phenolic acids—favourably modulate gut microbiota by increasing *Bifidobacterium* levels. This highlights their potential role in the development of targeted dietary interventions or supplements aimed at improving gut health and preventing or managing related metabolic or inflammatory disorders. However, given the substantial heterogeneity among existing studies and the limited number of high-quality randomised controlled trials, future research should prioritise well-designed, large-scale studies using standardised methodologies. Although polyphenols have long been recognised for their ability to influence gut microbiota and host physiology, much of the current evidence is inconsistent and derived from short-term interventions. Long-term trials conducted across diverse populations, complemented by multi-omics approaches (such as metagenomics, metabolomics, and transcriptomics), could provide deeper mechanistic insights into how polyphenols shape microbiota function and influence host health. Additionally, exploring personalised nutrition strategies—particularly those accounting for individual variability in polyphenol metabolism and dose–response dynamics—may support more tailored and effective dietary recommendations.

## 5. Conclusions

This systematic review and meta-analysis examined the effects of dietary polyphenols on gut microbiota composition, with particular emphasis on *Bifidobacterium* abundance. Following PRISMA guidelines, the review synthesised evidence from recent human studies, and a meta-analysis was conducted using R (version 4.4.1) with metafor *and* meta packages. A random-effects model was applied to account for between-study variability, and standardised mean differences (SMDs) were calculated. Across the reviewed literature, polyphenols—including flavonoids, phenolic acids, and anthocyanins from foods such as berries, grapes, and green tea—were frequently associated with increases in beneficial gut bacteria such as *Bifidobacterium*, *Anaerostipes*, *Faecalibacterium prausnitzii*, and *Lactobacillus*, while reducing potentially harmful species. These microbial shifts were associated with improvements in metabolic outcomes, including enhanced lipid profiles, better insulin sensitivity, reduced inflammation, and improved intestinal integrity. The meta-analysis of four eligible studies demonstrated a significant positive effect of polyphenol intake on *Bifidobacterium* levels, with a pooled SMD of 0.81 (95% CI: 0.18–1.44, *p* = 0.0114), representing a moderate to large effect. Despite this, substantial heterogeneity was present (*I*^2^ = 77.4%), likely due to variations in polyphenol type, dosage, intervention duration, population characteristics, and microbiome assessment methods. These findings align with broader evidence showing that polyphenols exert beneficial modulatory effects on gut microbial ecology, though results remain influenced by individual metabotypes and methodological differences across studies. Overall, this study suggests that dietary polyphenols may act as modulators of gut health, with potential applications in preventing or managing chronic diseases linked to gut dysbiosis. However, significant gaps remain, particularly regarding long-term effects, species-level microbial interactions, and personalised responses to polyphenol intake. Future research should prioritise well-designed, large-scale, long-duration clinical trials, employ standardised analytical protocols, and integrate multi-omics approaches to fully elucidate the mechanisms underlying polyphenol–microbiota–host interactions. Taken together, the findings reinforce the growing potential of polyphenol-rich diets and functional foods as effective tools for supporting gut and metabolic health.

## Figures and Tables

**Table 1:** PICO Framework. The review design was guided by PICO Framework.

Criteria	Determinants
Population	Human adults (≥18 years), both healthy and with conditions such as obesity or NAFLD
Intervention	Dietary polyphenols, including flavonoids, phenolic acids, stilbenes, and lignans
Comparison	Placebo, baseline, or control groups (where applicable)
Outcome	Changes in gut microbiota composition, microbial diversity, taxa abundance, and production of SCFAs

**Table 2:** Literature search strategy.

Keyword A (Polyphenols)	AND	Keyword B (Gut Microbiota)	AND	Keyword C (Clinical Context)
polyphenols	AND	gut microbiota	AND	human intervention
flavonoids	AND	intestinal flora	AND	clinical trial
dietary polyphenols	AND	microbiome	AND	randomized study

**Table 3:** PICO Framework for study selection.

PICO Element	Description
Population (P)	Human adults (≥18 years) exposed to dietary polyphenols, including both healthy individuals and those with metabolic or gastrointestinal conditions.
Intervention (I)	Consumption of polyphenols through natural food sources (e.g., berries, green tea, cocoa, red wine, nuts) or through supplements containing defined polyphenolic compounds such as flavonoids, phenolic acids, stilbenes, and lignans.
Comparator (C)	Placebo, baseline condition, or standard/control diet where applicable.
Outcomes (O)	Quantitative and qualitative changes in gut microbiome composition, including microbial diversity, shifts in specific microbial taxa, and alterations in microbial metabolites such as short-chain fatty acids (SCFAs).

**Table 4:** Inclusion and Exclusion Criteria.

Inclusion Criteria	Exclusion Criteria
Human intervention studies (e.g., randomized controlled trials, cohort studies)	Animal or in vitro studies only
Studies published in English between January 2013 and February 2025	Non-English publications
Interventions involving polyphenol-rich foods or supplements	Studies not involving polyphenols or mixed interventions where polyphenol effects could not be isolated
Outcomes assessing gut microbiota composition, microbial diversity, or metabolites such as SCFAs	Studies lacking measurable microbiota-related outcomes
Peer-reviewed, full-text articles	Abstract-only records, conference proceedings, editorials, or commentaries
Use of validated microbiota assessment techniques (e.g., 16S rRNA sequencing, shotgun metagenomics, or metabolomics)	Microbiota analysis not clearly described or lacking methodological transparency

**Table 5:** Summary the study intervention on dietary polyphenols and gut microbiota.

Author(s), Year	Study Design	Population	Polyphenol Type	Microbiome Assessment	Key Findings
Le Sayec et al., 2022 [13]	Double-blind RCT, 12-week	102 middle-aged prehypertensive adults	Aronia berry extract (capsule, 106 mg polyphenols)	Shotgun metagenomic sequencing	Increased gene richness and beneficial taxa; improved arterial stiffness; metabolic pathway enrichment.
Ntemiri et al., 2020 [14]	Mixed method (in vitro + pilot human study)	17 healthy women aged 21–77	Whole blueberry and isolated polyphenol fractions	16S rRNA sequencing (V3–V4), QIIME	In vitro and human shifts in *Faecalibacterium*, Anaerostipes; older group showed improved diversity.
Chamberlin et al., 2024 [15]	Double-blind RCT, 30-day	14 healthy adults aged 18–55	Aronia melanocarpa juice (300–320 mg polyphenols)	16S rRNA sequencing (V4), MOTHUR	Stabilised cholesterol and lowered glucose; reduced microbial richness; metabolic shifts observed.
Barnett et al., 2021 [16]	Randomised Controlled Trial	25 healthy adults	Red vs. white-fleshed apples (anthocyanin-rich)	16S rRNA sequencing	Reduced *Streptococcus*, *Ruminococcus*; increased *Lactobacillus*; altered immune gene expression.
Rinott et al., 2022 [17]	Randomised Controlled Trial	294 adults with abdominal obesity/dyslipidemia	Green-MED diet (walnuts, green tea, Mankai)	16S rRNA & Shotgun sequencing	Significant microbial shifts; enriched *Prevotella*; linked to cardiometabolic improvements.
Onali et al., 2025 [18]	Randomised Controlled Trial	43 healthy adults	Mixed berries with high meat diet	16S rRNA	Increased polyphenol metabolites; reduced colon cancer cell viability; protected microbiota profile.
Narduzzi et al., 2022 [10]	Review Article	Not applicable	(Poly)phenolic compounds	Not specified	Highlighted metabotypes influencing polyphenol efficacy; proposed personalised nutrition strategies.
Ramos-Romero et al., 2020 [19]	Randomised Crossover Trial	49 adults with cardiometabolic risk	Grape pomace (8 g/day)	qPCR	Minor microbiota changes; insulin reduction in responders; increased *Bacteroides* in non-responders.
Wood et al., 2023 [20]	Double-blind RCT	61 healthy older adults (65–80 years)	Wild blueberry (302 mg anthocyanins)	16S rRNA	Improved vascular and cognitive function; no gut microbiota changes.
Vetrani et al., 2020 [21]	Randomised Controlled Trial	78 adults at high cardiometabolic risk	Diets rich in polyphenols and/or long-chain omega-3 fatty acids	DGGE and qPCR	Increased microbial diversity; elevated CLEPT and reduced EREC; correlated with improved glucose and insulin responses.
Martínez-Montoro et al., 2022 [22]	Randomised Crossover Trial	20 adults (10 healthy and 10 with metabolic syndrome)	Phenolic-content beers (alcohol-free, lager, dark beer)	16S rRNA sequencing	Increase in *Streptococcaceae* and *Streptococcus* after dark beer consumption; reduction in porphyrin metabolism; effects influenced by metabolic status.
Wang et al., 2022 [23]	Review Article	Not applicable	Dietary polyphenols in general	Overview of in vitro and in vivo studies	Summarises mechanisms through which polyphenols modulate gut microbiota and are bio transformed into active metabolites by microbial activity.
Meir et al., 2021 [24]	Randomised Controlled Trial (DIRECT-PLUS)	294 adults with non-alcoholic fatty liver disease (NAFLD)	Green-MED diet with polyphenols from Mankai, green tea, and walnuts	Beta-diversity analysis and species-level profiling	Reduction in intrahepatic fat; higher plasma polyphenol levels; changes in gut microbiota linked to metabolic health improvements.
Ross et al., 2024 [25]	Narrative Review	Not applicable	Polyphenols in Mediterranean diet (e.g., flavan-3-ols)	Not specifically conducted	Gut microbiota may modulate polyphenol bioavailability and influence neurodegeneration and aging.
Catalkaya et al., 2020 [26]	Review Article	Not applicable	Various dietary polyphenols	In vitro and animal/human model references	Polyphenols are biotransformed by gut microbiota; this interaction enhances bioactivity and affects host health.
Zhang et al., 2022 [6]	Randomised Crossover Clinical Trial	36 adults (PreDM-IR and healthy controls)	Red raspberry (RRB) with or without fructo-oligosaccharides	Shotgun sequencing	Modulated gut microbiota composition: *Bifidobacterium* species increased; Ruminococcus gnavus decreased; associated with improved metabolic markers.
Meslier et al., 2020 [27]	Randomised Controlled Trial	82 overweight and obese individuals	Mediterranean diet rich in polyphenols	Shotgun metagenomics	Lowered plasma cholesterol and bile acids; microbial gene richness increased; *Faecalibacterium* prausnitzii abundance increased.
La Rosa et al., 2023 [28]	Review (in vitro, animal and clinical)	MS patients and preclinical models	Resveratrol, curcumin, luteolin, quercetin, hydroxytyrosol	Not primarily a microbiome study	Polyphenols exhibit anti-inflammatory and neuroprotective effects via microbiota-mediated mechanisms.
Peron et al., 2021 [29]	Randomised Controlled Crossover Trial	51 older adults (≥60 years)	Polyphenol-rich diet (cocoa, green tea)	16S rRNA profiling and metabolomics	Improved intestinal barrier function; serum polyphenol metabolites correlated with beneficial butyrate-producing bacteria.
Jinato et al., 2022 [30]	Randomised Double-Blind Placebo-Controlled Trial	38 patients with NAFLD	Oligonol (litchi-derived polyphenol)	16S rRNA sequencing	Liver steatosis improved; pathogenic bacteria reduced; short-chain fatty acid-producing bacteria increased.
Ntemiri et al. (2020) [14]	In vitro colon model and pilot clinical trial	17 women (pilot) and in vitro stool samples	Anthocyanins, proanthocyanidins, total polyphenols from blueberry	16S rRNA sequencing (in vitro and in vivo)	Blueberry fractions altered gut microbiota composition; alpha diversity increased in older adults; correlated with antioxidant activity.
Park et al., 2020 [31]	Randomised Controlled Trial	40 young adults with depressive symptoms	Flavonoid-rich orange juice (600 mg/day)	qPCR and sequencing of stool samples	*Lachnospiraceae* family and related taxa increased post-intervention; associated with improvement in depressive symptoms.

## Data Availability

No new data were created.
